# An exploratory study of factors associated with human brucellosis in mainland China based on time-series-cross-section data from 2005 to 2016

**DOI:** 10.1371/journal.pone.0208292

**Published:** 2019-06-14

**Authors:** Yun Lin, Minghan Xu, Xingyu Zhang, Tao Zhang

**Affiliations:** 1 Department of Epidemiology and Health Statistics, West China School of Public Health and West China Fourth Hospital, Sichuan University, Chengdu, Sichuan, China; 2 Applied Biostatistics Laboratory, University of Michigan School of Nursing, Ann Arbor, Michigan, United States of America; Faculty of Science, Ain Shams University (ASU), EGYPT

## Abstract

**Objective:**

Many studies focused on reasons behind the increasing incidence and the spread of human brucellosis in mainland China, yet most of them lacked comprehensive consideration with quantitative evidence. Hence, this study aimed to further investigate the epidemic mechanism and associated factors of human brucellosis so as to provide thoughts for future countermeasures in China and the rest of the world.

**Methods:**

Data of human brucellosis incidence and some associated factors in economy, animal husbandry, transportation as well as health and hygiene were collected at provincial level from 2005–2016. Time series plots were first used to visualize the annual incidence and annual rate of change of human brucellosis for each province, then cluster analysis categorized all the 31 provinces of mainland China based on their incidence time series during the study period. In addition, according to the characteristics of data, the dynamic panel data model in combination with supervised principal component analysis was proposed to explore effects of associated factors on human brucellosis.

**Results:**

1. The incidence rate of human brucellosis in mainland China increased three-fold from 1.41 per 100,000 people in 2005 to 4.22 per 100,000 people in 2014, though it went down a little in 2015 and 2016. Incidence rates in the north have always been higher than those in the south, but the latter also experienced an upward trend especially between 2012 and 2016. 2. The 31 provinces of mainland China were categorized into three clusters, and each cluster had its own characteristics of incidence time series. 3. The impact of health and hygiene situations on the prevention and control work of human brucellosis was still very limited and trivial (regression coefficient = -0.02). Therefore, it was plausible to presume that improving the personal average number of medical institutes and the proportion of rural medical expenditure might be helpful in preventing and controlling human brucellosis.

**Conclusions:**

The epidemic status of human brucellosis has changed in both spatial and temporal dimensions in recent years in mainland China. Apart from traditional control measures, more attention should be paid to the improvement of medical healthcare especially in rural areas in the hope of enhancing the control effect.

## Introduction

Human brucellosis is a highly contagious zoonotic disease mainly caused by unpasteurized milk or undercooked meat products that were made of infected animals. Direct contacts with ill animals can also cause brucellosis infection. On a global scale, brucellosis was and still is an important zoonosis across the world [1–3]. In China, it has been listed as the class B notifiable infectious disease since 2005 [4,5], as well as one of the most serious types of class B diseases among those listed in the *Detailed Rules for the Implementation of the Regulations on Livestock and Poultry Epidemic Prevention* [6]. The incidence of human brucellosis in mainland China decreased during the 1980-1990s but then rose steadily from 1995 till the 2014 peak. Apart from the temporal trend, the epidemic of human brucellosis in mainland China also had some spatial characteristics. Geographically, most of the northern provinces are pastoral and half-pastoral areas while most of the southern provinces are non-pastoral areas. Since the massive number of pastures increased local residents’ risks of exposure to infected animals or their products, brucellosis (both among humans and animals) was severest in north-eastern China in the past [7]. However, some recent studies [8,9] showed that human brucellosis epidemics were spreading from traditional high-incidence pastoral and half-pastoral areas in the north to low-incidence non-pastoral areas in the south. Such a rapid trend of increase and spread deserved much attention. Though the government has noticed the problem and some countermeasures have already been taken (e.g. the application of brucellosis vaccine and the setup of brucellosis prevention institutes) [10,11], the situation has not been optimized yet.

Many studies have tried to explore reasons behind the increased incidence, and there were some widely-accepted explanations [12], including the rapid development of husbandry, changes in the feeding mode of livestock, the frequent trading of livestock products among different areas, the increasing mobility of infected animals and so forth. In terms of the expansion of involved areas, previous study [13] assumed that people’s increasing opportunities to contact infected animals directly or indirectly in a wider area in recent years might be the reason. In the study of Jiang et al [14], it was found that the northern and southern *Brucella* strains shared the same MLVA-16 genotype, which verified another popular speculation that the epidemic in the southern area was partly caused by the import of infected animals from the other areas. Therefore, it could be summarized that the development of husbandry and trading was the most commonly accepted explanation for human brucellosis epidemics in recent years.

Although previous studies were instructive and illuminating for the prevention and control of human brucellosis, they still had two insufficiencies. First of all, there lacked comprehensive considerations of the multifactorial influences underlying the epidemics of human brucellosis. Both the increasing incidence rates and the rather poor performance of the prevention and control work of human brucellosis in recent years reminded us that there might be many factors interacting with each other and jointly influencing the incidence. However, some studies [4,7,8] only described the spatial and temporal characteristics of human brucellosis and did not involve building models for further quantitative analysis (such as exploration of associated factors); some [15] built the time series model of human brucellosis incidence but failed to penetrate its associated factors; another studies [16,17] considered associated factors only limited to a few aspects (such as environmental and animal husbandry factors, knowledge attitude practice, meteorological factors, etc). The second insufficiency was that most of these previous studies chose qualitative methods instead of quantitative ones. The former method was indeed the basic premise of inference; however, it could only be used to initially suggest the relations among different variables. Even with those very few quantitative studies [18, 19], they have only focused on a limited geographic area or a particularly small population, which weakened their ability in providing evidence for carrying out more effective human brucellosis countermeasures on a larger scale.

In recent years, the One Health campaign has attracted much attention. The One Health concept emphasizes that humans, animals and the ecosystem are interrelated with each other, and in order to tackle problems of infectious diseases (especially zoonoses), we need to advocate cooperation across different disciplines [20]. Therefore, in light of the current situation of human brucellosis prevention and control as well as the One Health approach, this study was the very first one aimed at assessing the spatial and temporal characteristics of human brucellosis incidence and the joint influences of different associated factors in mainland China in the last decade (2005 to 2016). Compared with previous studies, this study not only collected more comprehensive information but also used a more flexible model to obtain new knowledge from our acquired data. Specifically, standing on the aforementioned two insufficiencies of traditional studies, our work would make new contributions in the corresponding two ways.

### (1) More comprehensive information

The information of this study had three main features: 1. *wide spatial scale*. Our data covered 31 provinces in mainland China; 2. *long time period*. This study covered the years between 2005 and 2016, during which China had developed very rapidly in economy and society; 3. *multifaceted data source*. Our dataset covered a relatively large number of variables (13 in total) in relation to economic development, husbandry, transportation as well as health and hygiene. Though previous research [8, 15, 16] might collect more variables in one or two related fields than our study, no study ever has covered all aspects of these fields so comprehensively before. Such a wide range of data source would help to integrate information in an effective way and make the analysis results more comprehensive and more credible to some extent.

### (2) More flexible model

The analysis of this study confronted with two major challenges. The first challenge was the complicated spatial and temporal effects within the dataset which had violated the necessary independence assumption for the use of traditional regression models, while the second challenge lied in the relatively small sample size in related to the observations of each province, which would lead to large sampling errors and the lack of statistical power. To deal with both challenges, the dynamic panel data model combined with supervised principal component analysis (PCA) is used to quantify the effects of associated factors of human brucellosis. The dynamic panel data model compensated for the violation of independence by simultaneously modelling both the spatial and temporal effects [21]. Furthermore, supervised PCA can reduce original factors into fewer linear combinations with good representativeness of those original ones [22], which would, in turn, decrease the number of factors and remit the challenge of small sample size. To our knowledge, this was the first time that a combined model of dynamic panel data model and supervised PCA was applied to the study of human brucellosis, and because this combined model can solve these two commonly-seen problems in the brucellosis research data at the same time, it can be used more flexibly in practice.

Overall, the goal of this study was to analyze the spatial and temporal characteristics of human brucellosis incidence and to explore effects of its associated factors in a comprehensive and quantitative way, which would hopefully provide novel thoughts for making sophisticated and specific strategies for the prevention and control of human brucellosis in China and the rest of the world.

## Materials and methods

### Materials

Annual incidence rates of human brucellosis of the 31 provinces in mainland China from 2005 to 2016 were excerpted from the national internet-based reporting system of notifiable communicable diseases in China that was officially launched on January 1^st^, 2004. Besides, the annual rate of change for each province in each year was calculated as the difference of the incidence rates between the current year and the previous year. A total of 13 potential factors associated with human brucellosis were included from the China Health Statistical Yearbook (http://www.nhc.gov.cn/zwgk/tjnj1/ejlist.shtml) and the China National Knowledge Infrastructure (CNKI) website (http://www.cnki.net/). All these associated factors were collected on a per annum and per province basis, and detailed explanations of these factors were given in Table 1. These collected factors involved areas of economy and animal husbandry, transportation as well as health and hygiene, which took consideration of the influences of humans, animals and the environment. It should be noted that as prescribed by the *Law of the P*.*R*. *China on the Prevention and Treatment of Infectious Diseases* [23], suspected and confirmed cases must be reported to the local health and anti-epidemic agency within a specified time limit. Further, in accordance with the *Statistics Law of the P*.*R*. *China* [24], state organs, enterprises, public institutions and other organizations, as well as sole proprietorships and individuals shall provide truthful, accurate and complete data needed for statistical investigation in a timely manner. Such legal requirements guaranteed that biases such as underreporting both the incidence and risk factors in rural areas were well eliminated.

**Table 1:** Explanations for associated factors.

Factor name	Abbreviation	Meaning
Output value of animal husbandry (Hundred million Yuan) per 100,000 people	*animal_husbandry*	The value of all products of animal husbandry per capita, represented by money and all kinds of supportive services for animal husbandry production, which can reflect the scale of animal husbandry in a year.
Number of sheep (Ten thousand) per 100,000 people	*sheep_num*	The amount of sheep kept by all units and urban residents per capita at the end of the year.
Number of cattle (Ten thousand) per 100,000 people	*cattle_num*	The number of cattle kept by all units and urban residents per capita.
Mutton production (Ten thousand tons) per 100,000 people	*mutton_prod*	The weight of mutton that was butchered in that year per capita in the whole society.
Beef production (Ten thousand tons) per 100,000 people	*beef_prod*	The weight of beef that was butchered in that year per capita in the whole society.
Gross Domestic Product (Hundred million Yuan) per 100,000 people	*GDP*	The value of all final products and services produced by all permanent units in a country (or region) per capita for each year during the study period (2005–2016).
Turnover value of the whole society (Hundred million tons per kilometre) per 100,000 people	*goods_transfer*	The number of goods transported by all means of transportation multiplied by the corresponding distance in the whole society, calculated per capita.
Total length of highways (kilometres) per 10,000 square kilometres	*highway*	The spatial average length of highways in the district.
Number of medical institutions per 100,000 people	*institute_num*	The average number of all licensed medical institutions in the area.
Number of health personnel (Ten thousand) per 100,000 people	*health_personnel*	The average number of all employees working in hospitals, primary medical-care institutions, public health institutions and other medical institutions.
Public health expenditure (Hundred million Yuan) per 100,000 people	*health_input*	The average financial allocation by governments at all levels for health undertakings.
Urban medical expenditure proportion (%)	*urban_medical_prop*	Medical and health care expenditure of urban residents as a percentage of consumption expenditure.
Rural medical expenditure proportion (%)	*rural_medical_prop*	Medical and health care expenditure of rural residents as a percentage of consumption expenditure.

Furthermore, all the factors could be categorized into three groups:

1. Type I (the economy and animal husbandry factors): *animal_husbandry*, *sheep_num*, *cattle_num*, *mutton_prod*, *beef_prod* and *GDP*.
2. Type II (the transportation factors): *goods_transfer* and *highway*.
3. Type III (the health and hygiene factors): *institute_num*, *health_personnel*, *health_input*, *urban_medical_prop* and *rural_medical_prop*.

### Methods

For the convenience of analysis and interpretation, all variables in Table 1 were standardized beforehand. If there is no extra explanation, names of the variables in the rest of this paper referred to the standardized variables rather than the original ones. In this section, spatial and temporal characteristics of human brucellosis epidemics in mainland China from 2005 to 2016 were initially described. Afterwards, the cluster analysis was used to categorize different geographic regions based on incidence time series of each province. Both the data description and cluster analysis could provide some preliminary insights into the spatial and temporal distribution of human brucellosis in mainland China. Furthermore, the exploratory analysis was conducted to explore the effects of exogenous associated factors (such as economy, animal husbandry, transportation as well as health and hygiene) on human brucellosis.

In the exploratory univariable model analyses, the basic form of dynamic panel data model (without supervised PCA) was shown in Eq (1):

$$y_{n,t}=\beta y_{n,t-1}\text{+}\gamma x_{n,t}+\lambda_{t}+\mu_{n,t}\;(n=1,2,\ldots ,N;t=1,2,\ldots ,T),$$ (1)

where *y*_*n*_,_*t*_ represented the human brucellosis incidence rate of the *n*-th province in the *t*-th year, and *y*_*n*_,_*t*-1_ referred to that in the past year. In addition, *x*_*n*_,_*t*_ was the value of the associated factor of the *n*-th province in the *t*-th year, *λ*_*t*_ indicated the temporal effect of the year *t* and *μ*_*n*,*t*_ the random effect of the *n*-th province in the *t*-th year. Specifically, the regression coefficient *γ* referred to the magnitude of the potential effect of factor *x* on human brucellosis incidence, and this coefficient was the parameter of interest for this study.

Furthermore, as mentioned in the introduction part, to simultaneously deal with both challenges of non-independence and small sample size, we combined the supervised PCA with the dynamic panel data model for the exploratory analysis. Factors in Table 1 were first selected based on their standardized univariable dynamic panel data regression coefficients on brucellosis incidence. Only factors with sufficiently large regression coefficients were allowed to enter the next step, where the allowance threshold was determined by the cross-validation method. Subsequently, for each type of factor, we calculated their principal components as the linear combinations of those selected factors, where the linear combination coefficients were computed by the eigenvector-based method. For example, *economic*_1_, *economic*_2_,…, and *economic*_*R*_ were used to represent the principal components of the type I factors (the economy and animal husbandry factors), where the value of *R* was determined by the cumulative contribution rate of the corresponding principal components. The cumulative contribution rate ranged from 0 to 100%, and the higher it was, the more eligible those principal components would be to represent those original factors. For type I factors, the cut-off point of the cumulative contribution rate was set to be 90%, which meant that the *R* principal components of the type I factors should at least contain 90% of the original information. Similarly, let *S* and *M* be the number of principal components for the type II factors (the transportation factors) and the type III factors (the health and hygiene factors), respectively, and the corresponding principal components can be denoted as *transfer*_1_, *transfer*_2_,…, and *transfer*_*S*_, as well as *health*_1_, *health*_2_,…, and *health*_*M*_. The cut-off point of the cumulative contribution rate was set to be 80% for type II factors (since only two original factors were considered in this study) and 90% for type III factors. As a result, the specific dynamic panel data model with supervised PCA for this study was built as follows:

$$y_{n,t}=\beta y_{n,t-1}\text{+}\sum_{r=1}^{R}\omega_{r}\cdot economic_{n,t,r}+\sum_{s=1}^{S}\nu_{s}\cdot transfer_{n,t,s}+\sum_{m=1}^{M}\zeta_{m}\cdot health_{n,t,m}+\lambda_{t}+\mu_{n,t},$$ (2)

where *n* = 1,2,…,*N* and *t* = 1,2,…,*T*. Here *economic*_*n*,*t*,*r*_ are the value of the *r-*th principal component in economy and husbandry of the *n*-th province in the *t*-th year, with similar definitions of *transfer*_*n*,*t*,*s*_ and *health*_*n*,*t*,*m*_. In addition, *ω*_*r*_, *ν*_*s*_ and *ζ*_*m*_ are the average effect of increase per unit in each of these three principal components. Definitions of other factors were the same as those in Eq (1).

Throughout this study, all analyses were done in R 3.5.0, using R packages {stats}, {plm} and {ggplot2}, which were downloaded from the Comprehensive R Archive Network (CRAN) at http://cran.r-project.org/ and installed in advance. Cluster analysis was conducted by the complete-linkage clustering method with the R command hclust(), and dynamic panel data model was built by the R command plm().

## Results

### Descriptions of the spatial and temporal distribution

**Fig 1:**
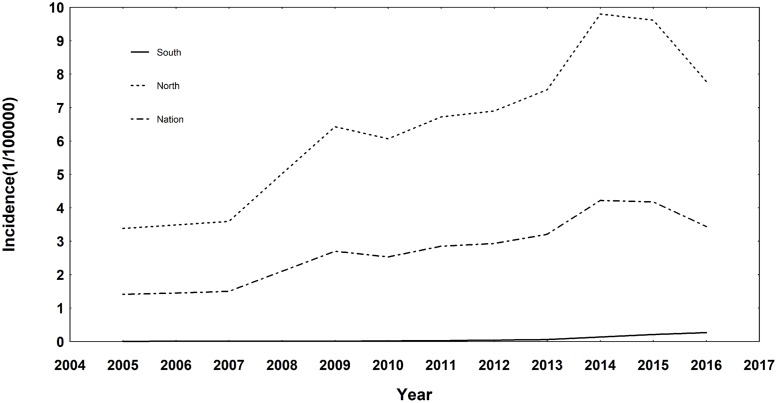
The time series plot of human brucellosis incidence. The time series plot of human brucellosis incidence in the whole country as well as in the southern and northern area of mainland China (result of the south-north partition could be seen in Fig 2, which was made based on the authoritative standard).

**Fig 2:**
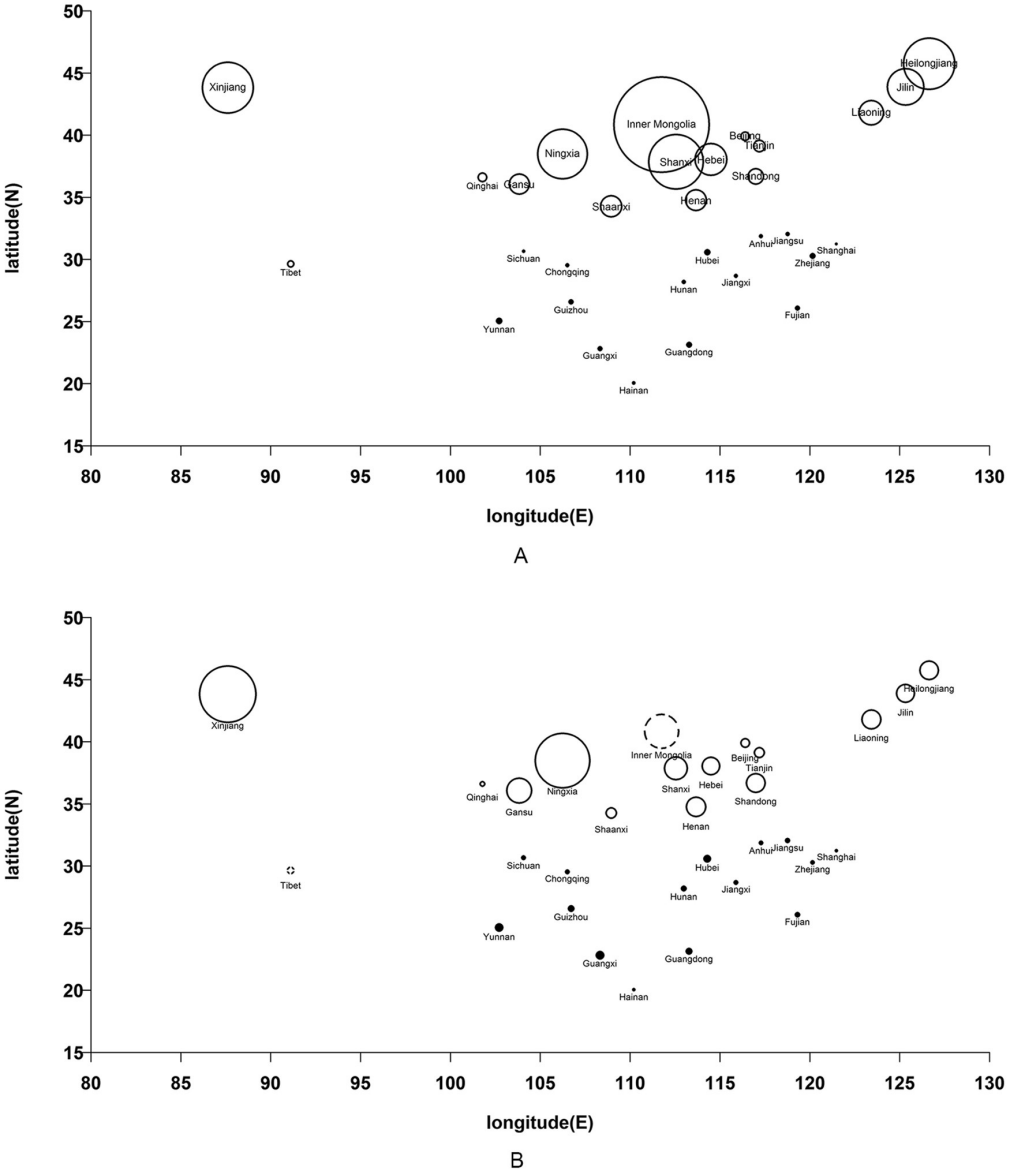
(A) the average incidence and (B)average annual rate of change of human brucellosis. The average incidence and average annual rate of change of human brucellosis in each province in mainland China, presented in (A) and (B) respectively. Each circle represented one province, where the horizontal and vertical coordinates of every circle’s center were the longitude (E) and latitude (N) of the corresponding province. The northern provinces were represented by hollow circles, and the southern provinces were represented by black circles. Specifically, in Fig 2B, since only Tibet and Inner Mongolia had negative average annual rates of change of human brucellosis, their corresponding circles were depicted by dashed lines; all the other provinces having positive annual rates of change were depicted by circles with solid lines. Furthermore, in Fig 2A, the area of the circle was proportional to the average incidence, and in Fig 2B, it was proportional to the absolute value of the average annual rate of change.

According to the time series plot of the nationwide human brucellosis incidence (Fig 1), the incidence rate increased three-fold from 1.41 per 100,000 people in 2005 to 4.22 per 100,000 people in 2014, though it went down a little in 2015 and 2016. With such an upward trend nationally, the epidemic situation also changed slightly in different regions. Though the northern incidence rate has always been higher than that in the south, which was in accordance with previous reports [25], the southern incidence also began to increase between 2012 and 2016 and such an increase even continued despite the decrease in the northern incidence since 2014. Furthermore, Fig 2 showed both the average incidence and the average annual rate of change of human brucellosis in each province of mainland China. From Figs 1 and 2, at least two points could be inferred: 1. the human brucellosis incidence rate went up significantly during the study period; 2. there was an overall upward trend of the epidemic in the south area while the northern provinces still kept high records of incidence rates. This indicated that the pastoral areas were still high epidemic areas while the incidence of human brucellosis also became more intense in half-pastoral areas and non-pastoral areas, which coincided with the conclusion of Zhang et al [26].

**Fig 3:**
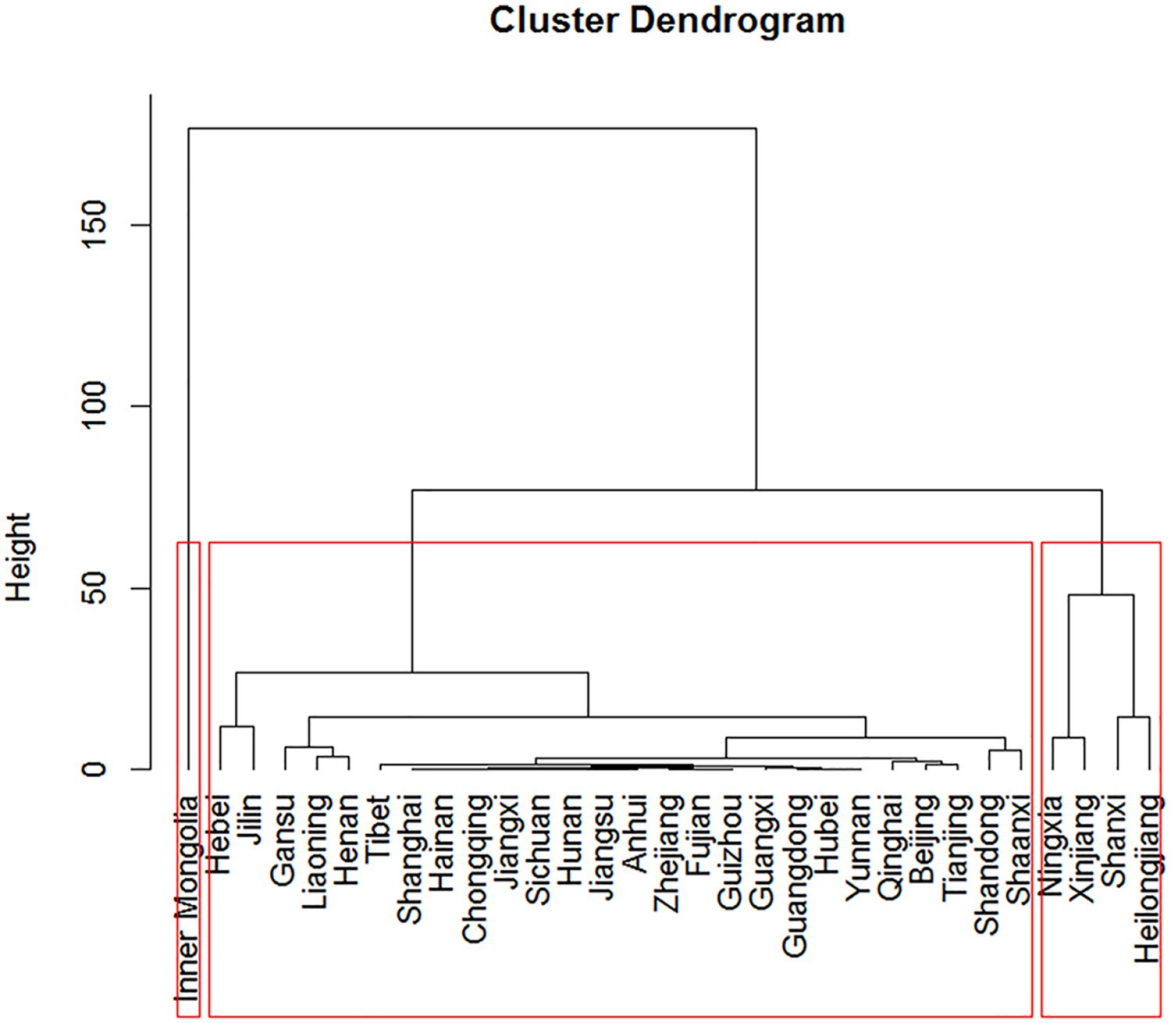
The cluster dendrogram of the 31 provinces. Each clade in the dendrogram represented one province in mainland China. The clades (provinces) were arranged according to how similar (or dissimilar) they were. Clades(provinces) of similar height were similar to each other; clades(provinces) of different heights were dissimilar. The red rectangle in the dendrogram showed that the 31 provinces in mainland China were categorized into three clusters.

**Fig 4:**
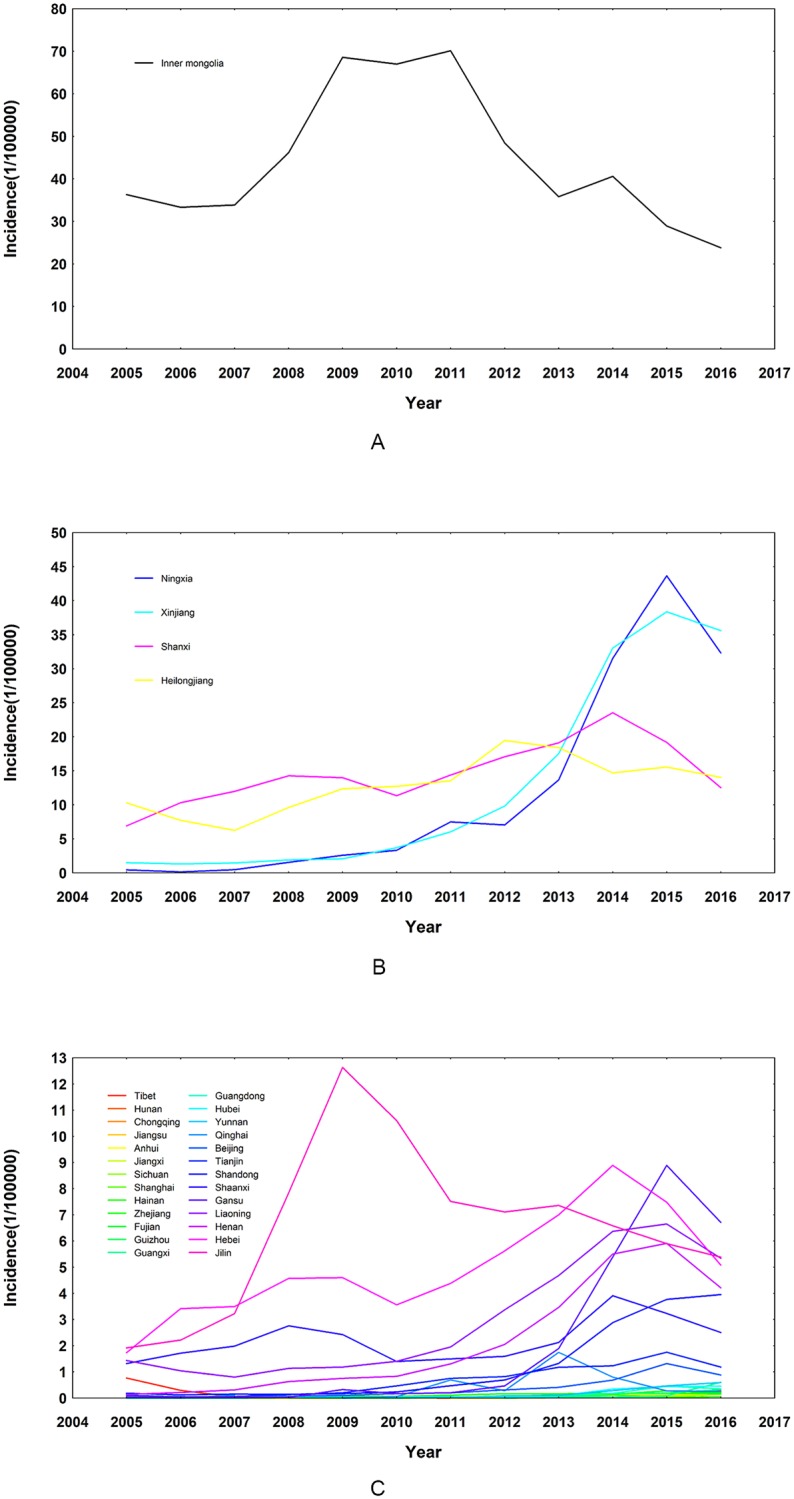
Time series plots of incidence rates for the three clusters. Time series plots of incidence rates for: (A) Cluster 1 (Inner Mongolia); (B) Cluster 2 (Shanxi, Heilongjiang, Ningxia and Xinjiang) and (C) Cluster 3 (the remaining 26 provinces in mainland China).

The clustering results were shown in Fig 3, which indicated that these 31 provinces could be classified into three clusters according to their incidence time series. Furthermore, based on the clustering results, time series plots of the three clusters were drawn respectively in Fig 4, which indicated that provinces in the same cluster did share similar shapes of time series plots. Specifically, Inner Mongolia alone belonged to Cluster 1. Its incidence rate has always been the highest in China with a steady increase since 2005; after reaching its peak in 2011, the rate dropped gradually despite a rebound in 2014. In addition, Ningxia, Xinjiang, Shanxi and Heilongjiang belonged to Cluster 2 and the remaining provinces were categorized into Cluster 3. For provinces in Cluster 2, their incidence rates were lower than that of Cluster 1, yet higher than most provinces in Cluster 3. Specifically, among the four provinces in Cluster 2, Ningxia and Xinjiang shared a more similar epidemic characteristic (peaking at 2015 after increasing steeply since 2011) while incidence rates of Shanxi and Heilongjiang remained steady at the level of 5–20 per 100,000 people most of the time. As for Cluster 3, it included 26 provinces such as Jilin, Tibet and Guangdong. These provinces had low incidence rates and mostly peaked during 2014–2015, but Jilin was an exception and experienced a decrease after an obvious rise in 2009.

Furthermore, since provinces in Cluster 2 (Shanxi, Heilongjiang, Ningxia and Xinjiang) and some provinces in Cluster 3 (Hebei, Liaoning, Shandong, Henan, Shaanxi and Gansu) all possessed a relatively high incidence rate and a similar shape of incidence time series, it was plausible to include them into further modelling analysis to find the underlying associated factors of human brucellosis. Other provinces were excluded from statistical modelling, for their extremely high or low incidence rates would perform as outliers and affect the validity and stability of statistical modelling. As a result, ten provinces in total were included in the statistical modelling stage, which were Gansu, Hebei, Heilongjiang, Liaoning, Henan, Ningxia, Shandong, Shanxi, Shaanxi and Xinjiang.

### Statistical modelling

Results of the dynamic panel data model with supervised PCA involved two parts: one was the determination of principal components, and the other was the estimation and interpretation of associated factors’ effects on human brucellosis.

#### Determination of principal components

Through calculation and comparison, 10 out of the 13 associated factors were selected as important associated factors, which were *GDP*, *animal_husbandry*, *sheep_num*, *mutton_prod*, *goods_transfer*, *highway*, *institute_num*, *health_personnel*, *health_input* and *rural_medical_prop*. The following principal components were then formed using these selected factors.

(1) Principal components of type I factors

The first two principal components were selected as representatives of type I factors since their cumulative contribution rate was 92.42%, and they were notated as *economic*_1_ and *economic*_2_ with the specific forms in Eqs (3) and (4).

$$economic_{1}=0.400\times GDP+\;0.373\times animal\_husbandry-0.608\times sheep\_num-0.576\times mutton\_prod,$$ (3)

$$economic_{2}=0.578\times GDP+0.605\times animal\_husbandry+0.352\times sheep\_num+0.421\times mutton\_prod.$$ (4)

From Eq (3), we could see that the direction of coefficients of *GDP* and *animal_husbandry* was different from the one of *sheep_num* and *mutton_prod*. Hence, we took *economic*_1_ as the differential factor related to the economy and animal husbandry. In contrast, the four coefficients in *economic*_*2*_ were in the same direction, so *economic*_2_ was taken as the common factor of economy and animal husbandry.

(2) Principal components of type II factors

Because there were only two principal components reflecting the situation of transportation and the cumulative contribution rate of the first principal component was only 70.7%, we included both of the two principal components into analysis, and they were notated as *transfer*_1_ and *transfer*_2_. Their equations were listed below:

$$transfer_{1}=0.707\times goods\_transfer+0.707\times highway,$$ (5)

$$transfer_{2}=-0.707\times goods\_transfer+0.707\times highway.$$ (6)

Similarly, coefficients of the two variables in *transfer*_1_ shared the same direction, and we called it the common factor reflecting the transportation situation; by contrast, *transfer*_2_ was taken as the corresponding differential factor.

(3) Principal components of type III factors

The first three principal components were selected as representatives of type III factors since their cumulative contribution rate was as high as 95.83%. They were notated as *health*_1_, *health*_2_ and *health*_3_ with the form in Eqs (7)–(9).

$$health_{1}=0.451\times institute\_num+0.542\times health\_personnel+0.536\times health\_input+0.464\times rural\_medical\_prop,$$ (7)

$$health_{2}=0.736\times institute\_num-0.675\times rural\_medical\_prop,$$ (8)

$$health_{3}=0.491\times institute\_num-0.341\times health\_personnel-0.563\times health\_input+0.571\times rural\_medical\_prop.$$ (9)

It could be seen from Eqs (7)–(9) that coefficient directions of the four variables in *health*_1_ were the same, so *health*_1_ was taken as the common factor reflecting the status of health and hygiene. In terms of *health*_*2*_, it implied the difference in coefficient directions of the two variables named *institute_num* and *rural_medical_prop*, meanwhile coefficients of *health_personnel* and *health_input* were estimated to be zero. Since *health*_*2*_ suggested the difference at the level of variable, it was regarded as the first-level differential factor of health and hygiene. Furthermore, *health*_*3*_ reflected the difference in the coefficient directions between two groups of variables (*institute_num*, *rural_medical_prop* versus *health_personnel*, *health_input*), thus it was regarded as the second-level differential factor of health and hygiene.

#### Estimation and interpretation of the associated factors’ effects

(1) The estimation results

The dynamic panel data model was built by including the aforementioned principal components and the incidence rate in the previous year (reflecting the temporal dynamic characteristics of the model) as associated factors. The goodness-of-fit results showed that the adjusted *R*^2^ of the model was 87.74%, which meant that the model could explain nearly 90% information of these factors’ effects on human brucellosis incidence. These goodness-of-fit results indicated that it was reasonable to conclude that the dynamic panel data model with supervised PCA could appropriately identify the effects of associated factors on human brucellosis. Table 2 showed the estimated coefficients in the model.

**Table 2:** The estimated coefficients of dynamic panel data model.

Associated factor	Coef	SE	*t*	*P*
*brucellosis*_*t*-1_	0.95	0.04	22.75	<0.0001
*economic*_1_	-0.35	0.29	-1.19	0.2378
*economic*_2_	-0.11	0.41	-0.26	0.7932
*health*_1_	-0.02	0.59	-0.03	0.9757
*health*_2_	-0.39	0.52	-0.76	0.4507
*health*_3_	-2.70	0.75	-3.62	0.0005
*transfer*_1_	-0.13	0.33	-0.40	0.6898
*transfer*_2_	-0.62	0.43	-1.44	0.1528

(2) The interpretation of results

Combined with the practice of human brucellosis prevention, the results could be interpreted as follows.

1. The human brucellosis incidence in the previous year was positively related to the incidence in the current year in the same place, which reflected the carry-over effect of brucellosis infection from one year to the next.
2. The coefficient of *economic*_2_ (the common factor) was -0.11, which indicated that economy and animal husbandry might possibly play an overall positive role in reducing risks of human brucellosis incidence. Conversely, the coefficient of *economic*_1_ (the differential factor) was -0.35. Combined with Eq (3) of *economic*_1_, we could assume that the increase of *sheep*_*num* and *mutton*_*prod* might lead to an increased incidence of human brucellosis (since their coefficients in the *economic*_1_ equation were -0.608 and -0.576 respectively), raising a presumption that the development of mutton industry might increase the epidemic risk of human brucellosis.
3. The coefficient of *health*_1_ (the common factor in health and hygiene) was -0.02, implying that the influence of health and hygiene situation on the prevention and control work of human brucellosis was still very limited and trivial. Results related to *health*_*3*_ in Table 2 indicated that improving *institute_num* and *rural_medical_prop* might be of great importance in the whole work of prevention and control. Furthermore, by inputting Eqs (7)–(9) into the dynamic panel data model in Table 2, the estimated regression coefficient of *institute_num* was -1.62, which indicated that there existed a possibility that the human brucellosis incidence could be reduced by 1.62/100,000 per one standard deviation increase in *institute_num*.
4. The regression coefficient of the common factor (*transfer*_1_) reflecting the situation in transportation was -0.13, hinting that development in transportation was more likely to be beneficial to the prevention and control of brucellosis. However, the absolute value of the regression coefficient of the differential factor (*transfer*_2_) was much larger than that of the common factor (*transfer*_1_), which implied that the influence of transportation on the incidence of human brucellosis might show more diversity than similarity. More underlying reasons would be discussed in the next section.

## Discussion

Human brucellosis is one of the few infectious diseases whose incidence rates still keep increasing nowadays in mainland China [27]. With the help of more comprehensive information and more flexible model than those of the previous studies, our work generated the following new knowledge and implications for the prevention and control work in this field, thereby helping to offer thoughts to make new prevention strategies.

Firstly, apart from the traditional recognition that human brucellosis incidence rates in northern China were much higher than those in the southern area, this study further analysed and compared the spatial and temporal epidemic characteristics of various areas. Specifically, according to the incidence time series, all provinces of mainland China were classified into three clusters, i.e., Cluster 1 with the highest incidence rate during the whole period of time, Cluster 2 with rather high incidence rates but lower than that of Cluster 1 and lastly, Cluster 3 whose incidence rates were at a comparatively low level.

Secondly, this study jointly considered some potential associated factors of human brucellosis which used to be considered separately in previous studies. Results indicated that the human brucellosis incidence rate in the prior year may be a risk factor for the current year’s possible epidemic (coefficient = 0.95). More importantly, results also implied that the number of medical institutes and rural medical expenditure proportion might be two factors that could be of great significance in the work of human brucellosis prevention and control.

Based on these new results, this study could provide new thoughts to the practical work of human brucellosis prevention and control in at least two ways:

1. Clustering could be used to better illustrate the heterogeneity and complexity of the transmission dynamics of human brucellosis in different spatial and temporal settings. This study classified the 31 provinces in mainland China into three clusters depending on the similar shape of incidence time series within the same cluster. Such partition could provide evidence for more effective countermeasures with greater pertinence targeting at various areas. Though previous study has tried to categorize areas in mainland China based on their different seriousness of epidemics, this classification was based on a very fixed standard, i.e., whether the human brucellosis incidence rates exceeded one case per million [28]; however, the fixed standard could not adapt to the rapid change of human brucellosis epidemics in China. In comparison, this study was more flexible and put it further by considering the incidence time series as the categorizing criteria. By doing this, researchers could not only get to know the severity of human brucellosis, but also further explore the spatial and temporal associations, know more about the epidemic mechanism as well as offer more practical clues for adjusting prevention measures in different conditions.
2. The exploratory analysis in this study contributed to indicating the potential effects of associated factors on human brucellosis in a more comprehensive way. It helped to update and deepen the understanding of epidemic mechanism as well as to locate the key areas of controlling human brucellosis more precisely. Here are some brief discussions on some factors involved.
  - **Historical impact** According to the analysis, there existed a statistically significant influence of the human brucellosis incidence rate of the previous year on the current incidence rate, which might be explained by the latency and invasiveness of *Brucella* [29].
  - **Transportation impact** Many scholars have assumed that the smuggling of infected animal products from other provinces might account for the human *Brucella* epidemics in those newly-emerging areas. In reality, however, the impact of transportation was quite complicated, which was in accordance with the estimated results of *transfer*_1_ and *transfer*_2_. The impact of transportation on the incidence of human brucellosis is complex in the sense that too much or too little development of traffic can both create suitable conditions for the epidemic of human brucellosis [30]. On the one hand, the development of tourism has promoted the flow of people, the booming of trade as well as the development of transportation in different regions. This may account for the spread of human brucellosis and the expansion of infection. On the other hand, human brucellosis is found to prevail in rural/nomadic communities where people live in close association with animals [31]. Those people who live in areas with poor transportation usually lack the awareness of human brucellosis. Besides, because of the remoteness of such places, there are fewer road transport quarantine stations, and the management of relevant departments is also poor (especially in terms of the veterinary services), which may cause an easier spread of the human brucellosis. This somehow explains why the regression coefficient reflecting the differential factor of transport (*transfer*_2_) is much larger than that of the common factor of transport (*transfer*_*1*_). The other point was that unlike common recognition, our analysis gave hint on a possible preventive influence of traffic development on human brucellosis, which could be found by inputting Eqs (5) and (6) into Table 2 and estimating the regression coefficients of *highway* on human brucellosis was -0.53. One potential explanation was that this study chose the average turnover value of the whole society (*goods_transfer*) and the spatial average length of highway routes (*highway*) to represent transportation, but in real life, the smuggling of animal products (especially those infected ones) tended to depend more on hidden routes instead of highways. The other possible explanation was that better transportation condition always associated with more advanced economic and social development, and in such cases, the quarantine and inspection measures and regulations would be more sophisticated and stricter, which would reduce the smuggling of infected animal products.
  - **Animal husbandry impact** Another widely-accepted opinion was that the development of husbandry might be the cause of human brucellosis [26], but no supporting result was found in this study. This reminded us that though there was a possibility that the development of husbandry created a more suitable environment for the epidemic of human brucellosis, some other factors might minimize or even counteract such a risk. However, it did not mean to deny the influence of husbandry development on human brucellosis, since human brucellosis is indeed a zoonosis, and the infection among humans always comes from infected animals [32]. By contrast, such a result emphasized that the risk created by the development of husbandry could be reduced if much attention could be paid to some protective factors of human brucellosis.
  - **Health impact** One interesting point of this study was that health and hygiene condition was associated with human brucellosis epidemics. More specifically, the result indicated that endeavor in increasing the number of medical institutes and in improving the rural medical expenditure proportion might bring benefits to the work of human brucellosis prevention and control.

The practical work of human brucellosis prevention and control involved allocating public resources in the most suitable and most cost-effective place especially when the budget is limited. Based on our analysed results, some advice could be proposed in perfecting the current work of human brucellosis prevention: (I) Governments should further enhance the communication and corporation among hospitals, township health centers, rural clinics, agricultural department and environmental department, as well as increase the investment in economy and infrastructure in medical institutes [33,34]. According to the One Health concept, brucellosis is a zoonosis, and in order to achieve optimal health outcomes, we should try to realize greater communication and collaboration among public health professionals, doctors and veterinarians. Moreover, considering that the rural and traditional pastoral areas are still high-risk regions, it is also recommended to pay more attention to the improvement of the quality of village-level public health service in the countryside, which helps to better carry out tertiary prevention among residents living there. (II) The prevention work can also be improved by raising the health awareness of rural residents and perfecting the new rural cooperative medical system [35]. Governments can increase the subsidized expenditure of health care in rural areas so as to maximize the possibility of an increase of the rural medical expenditure proportion. Since residents’ incomes can also affect their medical expenditure [36], current priority could be given to increase the health care subsidy of residents in these areas in order to achieve the goal of improving the medical expenditure proportion to control human brucellosis. To put it further, combined our result with the country’s most up-to-date policy and some related prevention campaigns [28], some advice could be proposed based on our results: to establish or enable more medical institutes for the detection and diagnosis of human brucellosis; to categorize different areas in mainland China based on their different epidemic situations of human brucellosis and utilize specific prevention strategies.

In today’s world, the prevention of human diseases involves a combination of human and other biotic factors, and the whole ecosystem. If we ignore this, we cannot go any further in improving the well beings of human. Hence, we need to pay enough attention to enhancing the cooperation among human, animals and the environment. However, though many people understand such a point, human-animal-environment collaboration lacks institutional and legal safeguards in terms of implementation, which weakens the role the whole system could play in solving real-life public health problems. Hereby, more attention should also be paid to establish a better communication mechanism across government and departments as well as to change passive monitoring into active monitoring. By doing this, we could give better play to the role of the environment in disease prevention and control.

Outside mainland China, many other regions in the world also suffer from human brucellosis, such as Brazil, Portugal and South Asia [37,38,39]. Although China is a country large in land and diverse in population composition, which could enhance the value of this study in providing evidence for better control measures in other areas, it is possible that different countries may have different problems with human brucellosis epidemics. On this basis, further studies with cross-national data and more associated factors are expected to contribute to faster and better prevention and control measures of human brucellosis worldwide.
